# Longitudinal analysis of physical function in older adults: The effects of physical inactivity and exercise training

**DOI:** 10.1111/acel.13987

**Published:** 2023-09-08

**Authors:** Kenneth M. Manning, Katherine S. Hall, Richard Sloane, Daniele Magistro, Emanuela Rabaglietti, Cathy C. Lee, Steven Castle, Teresa Kopp, Jamie Giffuni, Leslie Katzel, Michelle McDonald, Miles Miyamoto, Megan Pearson, Stephen C. Jennings, Janet Prvu Bettger, Miriam C. Morey

**Affiliations:** ^1^ Geriatric Research, Education, and Clinical Center VA Health Care System Durham North Carolina USA; ^2^ Department of Medicine, Center for the Study of Aging/Claude D. Pepper Older Americans Independence Center Duke University Medical Center Durham North Carolina USA; ^3^ Department of Orthopedic Surgery Duke University Medical Center Durham North Carolina USA; ^4^ Nottingham Trent University Nottingham UK; ^5^ Department of Psychology University of Turin Torino Italy; ^6^ Geriatric Research, Education, and Clinical Center VA Greater Los Angeles Healthcare System Los Angeles California USA; ^7^ David Geffen School of Medicine at UCLA Los Angeles California USA; ^8^ VA Medical Center Canandaigua New York USA; ^9^ Geriatric Research, Education and Clinical Center VA Maryland Health Care System Baltimore Maryland USA; ^10^ School of Medicine University of Maryland School of Medicine Baltimore Maryland USA; ^11^ Geritaric Rehabilitation and Clinical Center VA Pacific Health Care System Honolulu Hawaii USA; ^12^ Roybal Center Duke University Medical Center Durham North Carolina USA

**Keywords:** clinical, cohort, physical activity, physical performance, sedentary

## Abstract

Lack of exercise contributes to systemic inflammation and is a major cause of chronic disease. The long‐term impact of initiating and sustaining exercise in late life, as opposed to sustaining a sedentary lifestyle, on whole‐body health measures such as physical performance is not well known. This is an exploratory study to compare changes in physical performance among older adults initiating exercise late in life versus inactive older adults. Data from two observational cohorts were included in this analysis, representing two activity groups. The Active group cohort comprises older adults (*n* = 318; age 72.5 ± 7.2 years) enrolled in a supervised exercise program, “Gerofit.” The inactive group comprises older adults (*n* = 146; age 74.5 ± 5.5 years) from the Italian study “Act on Ageing” (AOA) who self‐reported being inactive. Participants in both groups completed physical performance battery at baseline and 1‐year including: 6‐min walk test, 30‐s chair stand, and timed up‐and‐go. Two‐sample *t*‐tests measured differences between Gerofit and AOA at baseline and 1‐year across all measures. Significant between‐group effects were seen for all performance measures (*p*s = 0.001). The AOA group declined across all measures from baseline to 1 year (range −18% to −24% change). The Gerofit group experienced significant gains in function for all measures (range +10% to +31% change). Older adults who initiated routine, sustained exercise were protected from age‐related declines in physical performance, while those who remained sedentary suffered cumulative deficits across strength, aerobic endurance, and mobility. Interventions to reduce sedentary behaviors and increase physical activity are both important to promote multi‐system, whole‐body health.

## Graphical abstract

The long‐term impact of initiating exercise in late life, opposed to sustaining a sedentary lifestyle, on health measures such as physical performance is not well known. This work observes these outcomes across two such cohorts and highlights the vast differences in performance longitudinally. Those who initiated sustained exercise were protected from substantial age‐related declines in strength, aerobic and endurance and mobility. Interventions to reduce sedentary behaviors and increase physical activity are both important to promote multi‐system, whole‐body health.

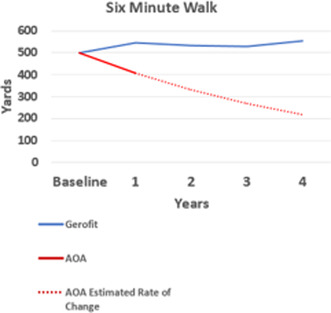

## 1 INTRODUCTION

Physical inactivity is associated with a host of problems later in life including impaired immune function, increased likelihood of chronic disease, loss of independence, decreased mobility and function, and early mortality (Burini et al., 2020; Carlson et al., 2018; Marques et al., 2014; Patterson et al., 2018; Silva et al., 2020; Visser et al., 2005). After age 70, aerobic capacity declines by greater than 20% over the subsequent 10‐year span (Fleg et al., 2005). Similar trends are reported for muscular strength (Frontera et al., 2000). Accelerated declines in cells and tissues underlie system‐level changes, such as cardiorespiratory and musculoskeletal systems, contributing to clinical risk factors including whole‐person physical function and maintenance of independence.

Gait speed and other physical performance measures are recognized valid predictors of important geriatric‐focused health outcomes including hospitalization, mobility disability, and institutionalization (Guralnik et al., 1989, 1995; Rikli & Jones, 2013). Rikli and Jones put forth a conceptual framework in which specific functional measures, validated against fitness tests, would identify modifiable capabilities across multiple domains of physical function (Rikli & Jones, 1999; Rikli & Jones, 2013). For example, the six‐minute walk test (6MWT) validated against treadmill testing, and 30‐s chair stand test validated with tests of knee extension and flexion. The battery of tests included age and gender‐based norms for functional assessments of strength, endurance, flexibility, and balance (Rikli & Jones, 1999, 2013). This framework has been widely adopted (Morey et al., 2018) with the addition of other performance measures prominent in geriatrics (Bohannon et al., 1996; Guralnik et al., 1994). With these assessments, exercise prescriptions could be targeted to specific modifiable functional deficits (Häkkinen et al., 2002; Magistro et al., 2014) and followed over time.

Exercise is a known lifestyle intervention that has shown antiaging effects by extending lifespan and healthspan (Carapeto & Aguayo‐Mazzucato, 2021). Limited information is available regarding effects of exercise on maintaining/improving physical function over an extended follow‐up in older adults. The majority of studies published have involved short, term‐limited interventions (e.g., 12 weeks, 6 months), and few include more than one follow‐up timepoint of physical function (Pahor et al., 2014). The purpose of this study is to examine the impact of exercise over the longer term in older adults, using a comprehensive functional fitness battery. To do this work, we needed to identify cohort(s) that included: (1) older age participant sample, (2) defined activity intervention and comparator group(s), and (3) recurrent, longitudinal functional assessments (12 months or longer follow‐up). We were unable to identify an existing cohort that met all of these criteria. Instead, we identified two separate cohorts, that together, provided the information that was needed for analysis. The two cohorts are “Gerofit” and “Act on Ageing” (AOA). Gerofit is a clinical exercise program for older veterans in the U.S. (Morey et al., 2018). Notable program elements are that participation in Gerofit is entirely voluntary and there is no term limit to participation, with many older adults participating for several years. Many participants feature multiple comorbidities associated with aging such as arthritis, cardiac and metabolic disease, and chronic pain. The AOA is an observational, prospective cohort study of sedentary older adults in Italy (Magistro et al., 2014, 2015). Both cohorts used the same physical performance battery, allowing us to compare functional trajectories over 1 year among older adults who sustained an exercise program (Gerofit) and those who remained inactive (AOA). An objective of this work is to test the hypothesis that engaging in a long‐term structured exercise program in late life prevents age‐related declines in physical function.

## 2 METHODS

### 2.1 Design and participants

#### 2.1.1 Cohort 1: Gerofit

Gerofit is a supervised, outpatient exercise program for older (65 years+) veterans offered by the Veterans Health Administration (VHA). It includes up to 3 days/week of exercise consisting of aerobic endurance, upper and lower body strengthening, balance and flexibility training. To qualify, participants must be in stable health, little/no cognitive impairment, independently mobile (use of assistive devices ok), and independent in activities of daily living.

#### 2.1.2 Cohort 2: Act on ageing

The AOA prospective cohort consists of sedentary, community‐dwelling older adults in the Piedmont area of Italy. To be eligible for the study, participants reported no regular participation in moderate‐intensity exercise in the 5 years prior to enrollment. Other eligibility criteria included cognitively unimpaired, able to walk independently (no use of mobility aides), with no history of cardiac surgery within the 12 months prior to study enrollment, free of uncontrolled hypertension or diabetes, and with no orthopedic fractures or impairments in the 6 months prior to enrollment (Magistro et al., 2015). With the exception of the mobility aides, inclusion criteria are highly similar across both cohorts.

### 2.2 Measures

Physical performance assessments were completed in both groups at 3 timepoints: enrollment, 6 months, and 1 year. All assessments were completed by trained staff using standard protocols.

*Six‐Minute Walk Test* (*6MWT*; Rikli & Jones, 2013): The total distance walked in 6 minutes was measured as an indicator of aerobic endurance.

*30‐Second Chair Stand Test*: The number of chair stands completed in 30 s was measured as an indicator of lower extremity strength (Rikli & Jones, 2013).

*Up&Go Test*: Different protocols for the Up&Go test were used across the two groups. In the Gerofit cohort, the 8 Foot Up‐and‐Go protocol was used. This measures the time it takes to rise from a chair, walk as quickly as possible up to and around a cone placed 8 feet from the chair, and return to a seated position (Rikli & Jones, 2013). In the AOA group, the Timed Up and Go (TUG) protocol was used (Podsiadlo & Richardson, 1991). This measures the time it takes to rise from a chair, walk at their usual walking pace up to and around a cone placed 3 meters from the chair, and return to a seated position. Both tests are reliable indicators of mobility and dynamic balance. Because there were slight differences in how the Up&Go tests were assessed, all comparisons between groups for these measures are described as percent change.

### 2.3 Statistical analysis

Gerofit is a national program with several locations across the U.S. Data for this analysis were gathered from four sites (Durham, NC; Baltimore, MD; Canandaigua, NY; and Los Angeles, CA). Participants included for analysis were individuals with a baseline, 12‐month, and least two follow‐up assessment time points up to 4 years, indicative of continued program attendance. This is consistent with previous Gerofit manuscripts to define individuals that were considered program adopters (Morey et al., 2018). To avoid contamination with the impact of COVID‐19 pandemic on program outcomes, these data are limited to 2014–2019.

*Primary aim*: One‐year changes in physical function were compared between Gerofit and AOA cohorts. We calculated the following for the three performance tests (6MWT, Chair Stands and Up&Go): the absolute change from baseline to 1 year (Year 1‐baseline) and the percent change ([Year 1‐baseline/baseline] × 100) over 1 year. The difference between the groups for change in these performance measures was tested using the t‐test. All statistical testing was conducted using SAS v9.4, with a *p*‐value of 0.05 used to determine significance.

*Secondary aim*: Subgroup change by cohort was evaluated by age group at baseline (65–74, 75–84, 85+) using Ordinary Least Squares (OLS) regression. Potential cohort by age group interactions and main effects of age group and cohort were assessed. These age groupings are consistent with classifying subjects as “youngest old” (65–74), “medium old” (75–84), and “oldest old” (85+) (Brown et al., 2022; Lee et al., 2018).

*Exploratory aim*: In the Gerofit cohort only, physical performance tests were completed annually up to 4 years. For illustrative purposes, the AOA baseline to 1‐year physical performance measure trajectories were extrapolated by extending the observed “baseline to 1 year” percent change for each year (i.e., Year 1 to Year 2, Year 2 to Year 3, and Year 3 to Year 4) to 4 years and plotted against the observed values for the Gerofit group.

This report is a retrospective study of quality improvement indicators of a clinical program. The Durham VA maintains an Institutional Review Board approval for retrospective analyses of the program which is reviewed and approved annually. Results are reported following the Standards for Quality Improvement Reporting Excellence (SQUIRE) reporting guidelines (Ogrinc et al., 2015).

## 3 RESULTS

### 3.1 Sample characteristics

The baseline Gerofit cohort consisted of 318 older adults, with an average age of 72.5 years (SD = 7.19), and BMI of 30.6 kg/m^2^ (SD = 5.32). The sample was predominantly male (94.6%), and evenly split between African American (50.3%) and Caucasian (49.7%). Additional descriptive characteristics of the cohort have been reported previously (Morey et al., 2018).

The AOA cohort consisted of 146 older adults, with an average age of 74.4 years (SD = 5.49) and BMI of 26.9 kg/m^2^ (SD = 3.88). All participants identified as Caucasian, and male.

### 3.2 Change in physical performance over 1 year

Table 1 displays the 1‐year change in both cohorts for the three outcome measures. On average, the Gerofit cohort improved over the 1‐year period while the AOA cohort declined in performance across all 3 functional measures. Using percent change as the analytic metric, the cohort differences were significantly different at *p* < 0.0001 for all three outcomes.

**TABLE 1:** Group means and differences between group means at 1 year.

Assessment	Active Gerofit (*n* = 318)				Sedentary‐act on ageing group (*n* = 145)				Between‐group differences
	Baseline	12 month	Change	Percent change	Baseline	12 month	Change	Percent change	
	M (SD)	M (SD)	M (SD)	M (SD)	M (SD)	M (SD)	M (SD)	M (SD)	
Six‐Minute Walk^a^ (year days)	500.8 (136.6)	544.9 (150.6)	44.1 (77.9)	10.9% (25.1)	460.7 (79.1)	377.7 (116.9)	−83.0 (69.7)	−18.7% (17.2)	*p* < 0.0001
Chair Stands^a^ (#)	12.0 (4.7)	15.1 (5.9)	3.1 (4.3)	30.6% (47.4)	11.5 (2.1)	8.3 (2.7)	−3.2 (1.9)	−24.5% (14.3)	*p* < 0.0001
Up&Go^b^ (s)	7.4 (4.3)	6.8 (4.7)		−9.2% (43.1)	8.2 (1.0)	9.8 (2.0)		19.4% (21.0)	*p* < 0.0001

^a^ Higher indicates better performance.

^b^ Lower indicates better performance. Between‐group differences defined as differences in percent change from baseline to 12 months (Gerofit vs. AOA). Comparison of raw change score is not shown for Up&Go assessments due to differing protocol.

*Six‐Minute Walk Test* (*6MWT*): On average, over the 1‐year time the Gerofit cohort improved the 6MWT distance by 44.1 yards, representing a 10.9% increase. The AOA cohort declined by 75.8 yards for a −18.7% change from baseline.

*30‐Second Chair Stand*: The Gerofit cohort improved the number of chair stands over 1 year by an average of 3.1, for a 30.6% increase. The AOA cohort declined by an average of 3.2 chair stands, for a −24.5% change from baseline.

*Up&Go Tests*: The Gerofit cohort improved their Up&Go time by 9.2%, while the AOA cohort declined on average by −19.4%.

### 3.3 Effect of age on changes in physical performance over 1 year

The OLS regression showed that for both cohorts, the time × age group interaction term was not significant. Improvements in physical function were observed across all decades over age 65 from Baseline to 1 year.

### 3.4 Descriptive changes in physical performance over 4 years

**FIGURE 1:**
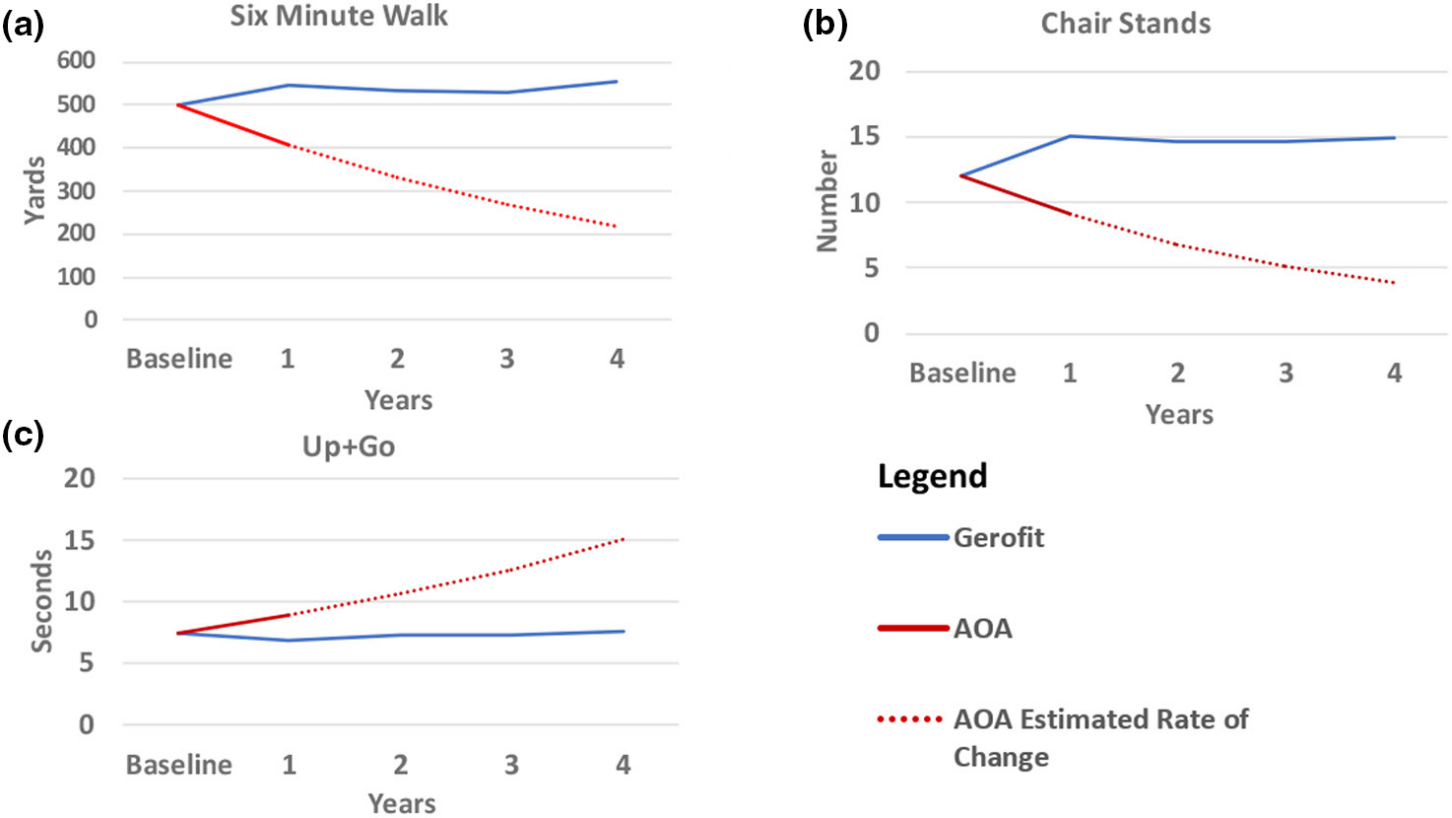
Physical function trajectories over time by cohort. All data shown for the Gerofit cohort are from observed physical performance assessments. Baseline and Year 1 data shown for the AOA cohort are from observed physical performance assessments; Year 2–4 data points are estimates.

Figure 1 illustrates the observed baseline and 1 year means by cohort for the three outcome measures. For the Gerofit cohort, the observed means at 2 (*n* = 140), 3 (*n* = 78), and 4 years (*n* = 40) are displayed. For the AOA cohort, the percent change from baseline to 1 year is carried forward for the Years 2, 3, and 4 to estimate trajectories.

On average, the Gerofit cohort maintains 1‐year improvements in the 6MWT, Chair Stands, and Up&Go over the 4 subsequent years.

*Six‐Minute Walk Test (6WMT*): In year two, the Gerofit cohort decreased 6MWT distance 13.4 yards, a 2.5% reduction from the previous year. In Year 3, the 6MWT distance decreased 1.9 yards, a <%0.1 change from the previous year. In Year 4, 6MWT distance increased 26.5 yards, a 4.8% increase from the previous year.

*30‐Second Chair Stand*. In Year 2, the Gerofit cohort decreased chair stands 0.41 stands from Year 1, 2.8% reduction. In Year 3, chair stands increased 0.02 chair stands, <0.1%. In Year 4, chair stands increased 0.25 chair stands, 1.7%.

*Up‐and‐Go Tests*: In Year 2, the Gerofit cohort increased (reflecting worsening performance) Up&Go time 0.49 s, a 6.6% increase from the previous year. In Year 3, Up&Go time decreased 0.01 s, a <0.1% change from the previous year. In Year 4, Up&Go times increased 0.23 s, a 3.0% worsening from the previous year.

### 3.5 Effect of age on changes in physical performance over 4 years

**FIGURE 2:**
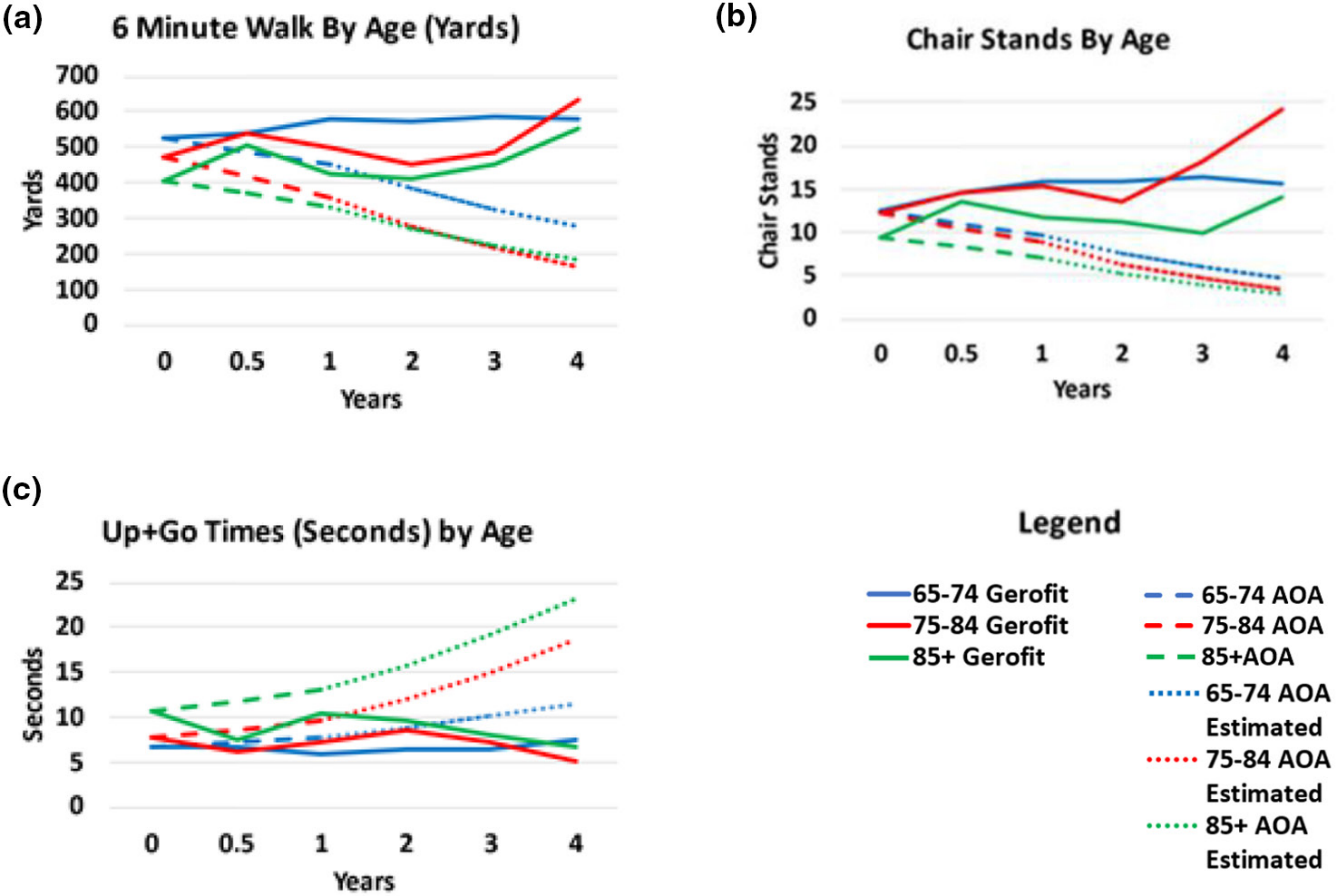
Physical function trajectories over time by age and cohort. All data shown for the Gerofit cohort are from observed physical performance assessments. Baseline and Year 1 data shown for the AOA cohort are from observed physical performance assessments; Year 2–4 data points are estimates.

Figure 2 illustrates the physical function data by decade of life from 65 years (65–74, 75–84, 85+) for each cohort, over 4 years. Improvements in physical function were observed across all decades over age 65 from baseline to 1 year.

## 4 DISCUSSION

The key findings of this study are the differences in 1‐year trajectories by activity status. The AOA sedentary cohort experienced a notable decline in function which stands in stark contrast to the Gerofit cohort who maintained and improved function following initiation of exercise. We have long sought to contextualize previously reported short‐term changes in physical performance in Gerofit, but this real‐world program lacks several attributes of a clinical trial, including a “non‐intervention” group. The comparison with the AOA cohort gives a compelling glimpse of age‐related changes at the person level with exercise, and the deleterious effects of physical inactivity over the longer term. Equally notable was that age did not matter; confirming the findings of others that benefits of exercise can be achieved even if initiated in late life, and further highlighting the detrimental impact of physical inactivity on healthy aging (Cidoncha‐Moreno et al., 2022; Moreno‐Agostino, 2020; Piercy et al., 2018).

Previous studies have documented performance cut points which indicate the strength, endurance, and mobility needed for maintaining physical independence in later life (Rikli & Jones, 2013). Both the AOA and Gerofit cohorts fell far short of the standards at baseline. When compared to age‐ and gender‐matched U.S. population norms (Rikli & Jones, 1999), baseline Gerofit participants ranked in the 13th percentile in endurance (6MWT), 24th percentile in lower body strength (chair stands), and < 5th percentile in balance and mobility (Up&Go). Despite these numbers, program participants were able to increase function across all measures and sustain improvements at levels greater than baseline values up to 4 years later. In contrast, the AOA group, a robust comparator of high‐functioning older adults with little health or disability burden, performance worsened. This report demonstrates the great potential for resilience in older age and the robustness of exercise as an intervention to attenuate age‐related declines in a sample with substantial health and function burden.

Preserving strength, endurance, and mobility is central to maintaining a high quality of life and independence in the community. Our findings contribute to a growing field of research testing exercise as a nonpharmacologic gerotherapeutic approach to prevent, delay, or attenuate functional decline and chronic disease (Forman et al., 2023; Grevendonk et al., 2021). Indeed, exercise as a transdiagnostic intervention tool that impacts biopsychosocial aspects of aging is acknowledged across disciplines and across the translational research spectrum. The molecular mechanisms by which exercise improves health are complex, and are currently under study in several NIH‐funded trials (Sanford et al., 2020). The Gerofit clinical program has amazing potential to fill this gap, with serial, longitudinal assessments of physical performance, and opportunities for ancillary efforts that combine laboratory‐based studies with this real‐world program (Ferrucci et al., 2016). Widespread implementation of the Gerofit program to other VHA medical centers is underway, offering even more opportunity for studying these associations and longitudinal trajectories in large, diverse sample of older adults with morbidity.

There are several strengths of this study. First, this study included longitudinal, repeated measures of physical performance in older adults. Much of the literature to date is rooted in epidemiologic studies that report cross‐sectional assessments of physical function by age (Cunningham et al., 2020; Paterson & Warburton, 2010; Ramsey et al., 2021). Second, the work described here was done in community‐dwelling older adults, in real‐world settings. And yet the findings reported here are consistent with reports from rigorously controlled clinical trials (Pahor et al., 2014). Third, the AOA is a robust comparator group, relatively free of chronic disease and functional burden; a stark contrast to the Gerofit sample. It can be safely assumed that the between‐group difference in physical performance over 1 year would have been even more pronounced if a sample with morbidity could have been identified. Finally, the diversity (racial and health profiles) of the Gerofit sample and the Gerofit program being embedded in the Veterans Health Administration, one of the largest coordinated care systems in the U.S. has implications for future implementation and dissemination of this intervention to sedentary older adults with morbidity.

This analysis is not without limitations. A limitation of the current report, but an area ripe for future study, is the inability to examine relevant pathways through which the benefits of exercise are conveyed on more holistic/whole‐person outcomes in the Gerofit cohort. Another limitation is missing data beyond 1 year. Caution is warranted when interpreting the 4‐year results, given our reliance on 4‐year estimated trajectories for this group and the inability to confirm activity status over that timeframe in the AOA comparator. Ideally, the AOA would have continued to collect repeated measures beyond 1 year. In the Gerofit cohort, the drop from 318 baseline assessments to *n* = 40 at 4 years limits our ability to draw any strong conclusions. Both attrition (drop‐out) and loss to follow‐up (still active in program but did not complete assessments) are at play here. For this project, we did not distinguish between these two types of loss of data, and acknowledge that the remaining data likely represent a “survival” bias with only the most robust individuals retained in the study. Additionally, factors such as a predominanly male sample, location (U.S. vs. Italy), BMI differences between cohorts, and lack of comorbidity data for the AOA sample warrant consideration when interpreting these findings.

There is a paucity of literature with longitudinal data using these physical performance assessments among sedentary older adults. The absence of longitudinal cohort studies with serial assessments of physical performance and no intervention led us to creatively integrate data from the Act on Ageing sedentary cohort. We quantify here the impact of initiating and sustaining physical activity on physical performance in later life, and show the cumulative negative impact of sustaining an inactive lifestyle. These findings in a community‐based, real‐world setting are consistent with other studies, the majority of which are highly‐controlled clinical trials. For example, Pahor and colleagues reported that major mobility disability over 2.5 years was significantly reduced in older adults who initiated a long‐term structured exercise program compared to those who did not (Pahor et al., 2014). These data support the call for action to promote physical activity among sedentary adults, in particular among older adults with morbidity (Langhammer et al., 2018; Piercy et al., 2018).

## 5 CONCLUSION

This work fills a gap in understanding the powerful impact of sustained exercise on the physical performance of older adults. Important longitudinal trajectories are revealed among both sedentary and exercising older adults by decade of life. These data contribute to the ever‐growing evidence of the importance of exercise in late life, and the substantial harm of a sedentary lifestyle on functional independence in late life.

This Gerofit cohort represents a diverse comorbid population. Individuals with conditions such as obesity, diabetes, hypertension, cardiovascular disease, pulmonary disease, cancer, arthritis, and others have benefitted from participation (Addison et al., 2019; Cowper et al., 1991; Morey et al., 1988, 1991, 1996, 2002; Pepin et al., 2020; Wilkins et al., 2021). We have demonstrated here the powerful effect of exercise training to slow down the age‐related progression of the disabling cascade, even in the context of chronic disease (Rebelo‐Marques et al., 2018). Future studies utilizing geroscience approaches would enable us to assess the impact of exercise training on biological aging and healthspan in this cohort. The model of assessing and treating functional deficits with progressive, structured exercise should be transferrable to other settings treating similar conditions affected by a lack of exercise.

## AUTHOR CONTRIBUTIONS

Kenneth M. Manning, Katherine S. Hall, Cathy C. Lee, Steven Castle, Teresa Kopp, Leslie Katzel, Jamie Giffuni, Teresa Kopp, Michelle McDonald, Miles Miyamoto, Stephen C Jennings, Janet Prvu Bettger, Megan Pearson, and Miriam C. Morey: implementation and dissemination of Gerofit program; data collection; concept, design, and review of manuscript. Kenneth M Manning, Kenneth M. Manning, Janet Prvu, Richard Sloane, Stephen C. Jennings, Daniele Magistro, and Miriam C. Morey: data collection, analysis, preparation, and review of manuscript, ER: data collection, analysis. All authors: final approval of manuscript submitted.

## FUNDING INFORMATION

Gerofit dissemination has been funded by the Veterans Health Affairs Office of Geriatrics and Extended Care Non‐Institutional Long Term Care Funding and Mentored Partnership program and the VHA Office of Rural Health. The Gerofit program has been locally supported by the Durham VA Geriatric, Research, Education, and Clinical Program, Drs. Morey and Hall and Mr. Sloane are supported in part by the Duke OAIC NIH/NIA AG028716. Part of these data were presented at the Gerontological Society of America Annual Meeting of 2020.

## CONFLICT OF INTEREST STATEMENT

All authors have no conflict of interest or commercial relationships to disclose.

## Data Availability

The data that support the findings of this study are available on request from the corresponding author. The data are not publicly available due to privacy or ethical restrictions.
